# Multidisciplinary total eradication therapy (TET) in men with newly diagnosed oligometastatic prostate cancer

**DOI:** 10.1007/s12032-020-01385-7

**Published:** 2020-06-10

**Authors:** D. K. Reyes, S. P. Rowe, E. M. Schaeffer, M. E. Allaf, A. E. Ross, C. P. Pavlovich, C. Deville, P. T. Tran, K. J. Pienta

**Affiliations:** 1 The James Buchanan Brady Urologic Institute and Department of Urology, Johns Hopkins University School of Medicine, Baltimore, MD USA; 2 The Russell H. Morgan Department of Radiology and Radiological Science, Johns Hopkins University School of Medicine, Baltimore, MD USA; 3 Department of Urology, Feinberg School of Medicine, Northwestern University, Evanston, IL USA; 4 Texas Urology Specialists, Mary Crowley Cancer Research, Dallas, TX USA; 5 Department of Radiation Oncology and Molecular Radiation Sciences, Johns Hopkins University School of Medicine, Baltimore, MD USA; 6 Department of Oncology, Johns Hopkins University School of Medicine, Baltimore, MD USA

**Keywords:** Oligometastatic prostate cancer, Total eradication therapy, Oligometastases

## Abstract

To evaluate the outcomes of total eradication therapy (TET), designed to eradicate all sites of visible cancer and micrometastases, in men with newly diagnosed oligometastatic prostate cancer (OMPCa). Men with ≤ 5 sites of metastases were enrolled in a prospective registry study, underwent neoadjuvant chemohormonal therapy, followed by radical prostatectomy, adjuvant radiation (RT) to prostate bed/pelvis, stereotactic body radiation therapy (SBRT) to oligometastases, and adjuvant hormonal therapy (HT). When possible, the prostate-specific membrane antigen targeted ^18^F-DCFPyL PET/CT (^18^F-DCFPyL) scan was obtained, and abiraterone was added to neoadjuvant HT. Twelve men, median 55 years, ECOG 0, median PSA 14.7 ng/dL, clinical stages M0—1/12 (8%), M1a—3/12 (25%) and M1b—8/12 (67%), were treated. ^18^F-DCFPyL scan was utilized in 58% of cases. Therapies included prostatectomy 12/12 (100%), neoadjuvant [docetaxel 11/12 (92%), LHRH agonist 12/12 (100%), abiraterone + prednisone 6/12 (50%)], adjuvant radiation [RT 2/12 (17%), RT + SBRT 4/12 (33%), SBRT 6/12 (50%)], and LHRH agonist 12/12 (100%)]. 2/5 (40%) initial patients developed neutropenic fever (NF), while 0/6 (0%) subsequent patients given modified docetaxel dosing developed NF. Otherwise, TET resulted in no additive toxicities. Median follow-up was 48.8 months. Overall survival was 12/12 (100%). 1-, 2-, and 3-year undetectable PSA’s were 12/12 (100%), 10/12 (83%) and 8/12 (67%), respectively. Median time to biochemical recurrence was not reached. The outcomes suggest TET in men with newly diagnosed OMPCa is safe, does not appear to cause additive toxicities, and may result in an extended interval of undetectable PSA.

## Introduction

In 1995, the term oligometastases (OM) was coined to describe a disease state existing between localized and widespread systemic disease [1], possibly biologically distinct and more amenable to therapy. However, without a specific diagnostic test, oligometastases, derived from ‘oligo’ meaning ‘few’, is currently a diagnosis of imaging. The most common definition, ≤ 5 sites of metastases, is an arbitrary number, accepted by most as the dividing line between oligo- and poly-metastatic disease. Despite the absence of both a consensus definition and any specific diagnostic test for OM, patients with newly diagnosed prostate cancer (PCa) and limited metastases are being treated by targeting the primary and metastatic sites, if not to cure OM cancer, then at least to possibly turn back the clock of the natural course of the disease.

Men newly diagnosed with PCa and distant metastases have an overall 5 year survival rate of 31% [2]. However, there is an increasing appreciation that men with metastatic disease should be further classified by disease burden, either oligometastatic or low tumor burden versus polymetastatic or high tumor burden, and that different treatment strategies should be developed for these clinical states.

In OM, or low-burden metastatic PCa, randomized studies support radiation to the primary site [3], metastasis directed therapy (MDT) [4, 5] and systemic therapies, including the antiandrogen abiraterone acetate, which when added to ADT further inhibits androgen synthesis [6, 7] and taxotere [8, 9]. Retrospective data, in conjunction with SEER database studies and a Munich Cancer Registry study, support prostatectomy in men with metastatic prostate cancer [10–13]. While all the above studies have shown improved outcomes, multimodal therapy may further improve outcomes by targeting all visible disease as well as micrometastases.

The ORIOLE trial [4] found that men with oligometastatic prostate cancer (OMPCa) (recurrent hormone sensitive PCa with 1–3 metastases on conventional imaging), treated with stereotactic ablative radiotherapy (SABR) as compared to observation, were less likely to have disease progression. In the post hoc analysis, the investigators were unblinded to the prostate-specific membrane antigen (PSMA) targeted ^18^F-DCFPyL PET/CT scan (^18^F-DCFPyL scan) imaging findings. In men where all PSMA avid lesions were treated, there was decreased risk of later metastases, which supports consolidative MDT. However, the investigators also pointed out that most men do not achieve an undetectable PSA after SABR, which suggests micrometastatic disease is still present.

O’Shaughnessy et al. published a pilot study of multimodality treatment to definitively treat known sites of disease in early metastatic prostate cancer [14]. 20% of the patients had an undetectable PSA and non-castrate levels of testosterone at 20 months, suggesting that a multidisciplinary approach to treat all sites of clinically evident disease is worth exploring in men with OM disease at diagnosis. Similarly, Parikh et al. [15] is enrolling men with new OMPCa into a Phase II trial to treat with both systemic and tumor-directed therapies.

In 2015, we utilized a prospective patient registry to follow men under treatment for newly diagnosed metastatic PCa, including oligometastatic prostate cancer (OMPCa). A series of patients underwent treatment to eradicate all clinically evident disease, including neoadjuvant and/or adjuvant chemohormonal therapy, definitive therapy to the primary lesion through radical prostatectomy or external beam radiation therapy, and metastasis directed therapy (MDT) using radiation to the metastatic lesions. The registry allowed for inclusion of real-world clinical practice. The introduction of the ^18^F-DCFPyL scan [16], with the ability to detect previously clinically undetectable disease [17], changed the definition of OM disease in those patients who received it. Meanwhile, the addition of abiraterone to androgen deprivation therapy (ADT) was found to increase survival in men with newly diagnosed, metastatic castrate sensitive PCa [6], therefore supracastration therapy was added into treatment plans. Finally, an increased incidence of docetaxel related neutropenia and neutropenic fever (NF) was noted, therefore the dosing regimen was modified.

Here we discuss and report outcomes in men who presented with newly diagnosed, treatment naïve OMPCa, and chose to be treated with total eradication therapy (TET)—defined as definitive therapy to the primary lesion (prostatectomy), adjuvant radiation therapy (aRT) to the prostate bed and pelvis as needed, MDT for oligometastases (radiation), and systemic therapy for micrometastases (neoadjuvant chemohormonal therapy and adjuvant hormonal therapy).

## Material and methods

### Patient Population

Men with newly diagnosed, untreated OMPCa were considered for TET, with the intent to eradicate all clinically evident and microscopic disease. In addition to the TET approach, patients were offered hormone therapy alone (discussed as a standard of care), hormone therapy plus radiation therapy, and any available clinical trial where entry criteria were met. Patients who elected TET, and had at least 2 years of follow-up, were included in this report.

Pretreatment criteria for TET included histologic confirmation of prostate cancer and ECOG performance status ≤ 1. None of the patients had prior systemic therapy for PCa, prior local therapy to a metastatic site, prior primary therapy (prostatectomy or radiation), abnormal end organ function, active cardiac disease, or a prior history of malignancy (unless in remission > 5 years). Hormone therapy started < 90 days before initiating TET was allowed. All patients provided written informed consent to be followed in the Registry trial as approved by The Johns Hopkins University School of Medicine Institutional Review Board [18].

### Oligometastatic prostate cancer staging

OM was defined as ≤ 5 metastatic lesions, including bone lesions and lymph nodes, as demonstrated on a ^99m^Tc-bone scan and a contrast-enhanced CT of the abdomen/pelvis. During the study period, the novel PSMA targeted ^18^F-DCFPyL scan [16], was intermittently available through several imaging research protocols and was incorporated into OM staging.

### Multidisciplinary treatment

#### Hormone therapy

ADT with a luteinizing hormone-releasing hormone (LHRH) agonist was initiated along with chemotherapy, for 1- or 2- years duration based on physician discretion. Bicalutamide (50 mg daily) was allowed prior to or at the start of LHRH therapy, for ≤ 1 month. During the study period, the addition of abiraterone and prednisone to ADT was found to increase overall survival in men with newly diagnosed, metastatic castrate-sensitive PCa [6]. Hence, abiraterone acetate (1000 mg/daily) and prednisone (5 mg daily) were given concurrently during chemotherapy, dependent on the patient’s insurance coverage or ability to pay out-of-pocket.

#### Chemotherapy

Docetaxel was given for up to six, 21-day cycles. Premedication included dexamethasone (8 mg) twice daily, beginning the day before chemotherapy, for 3 days. Clinical and laboratory assessments were performed at baseline, with each cycle, and 3 weeks post-chemotherapy. Dose reductions (up to 40% of the standard 75 mg/m^2^ dose) and treatment delays (up to 5 weeks from the standard 3-week interval) were allowed to mitigate and manage toxicities. Over time, a higher than expected incidence of chemotherapy-related NF was noted, and determined to be due to competition of metabolic pathways when initiating docetaxel and hormonal therapy simultaneously in castrate-sensitive patients [19]. This led to a revised docetaxel dosing regimen of four, 21-day cycles of docetaxel at 30 mg/m^2^ for cycles 1 and 2, and of 60 mg/m^2^ for cycles 3 and 4. In one patient, prostatic adenocarcinoma was mixed with small cell carcinoma (SCC) and cisplatin and etoposide (CIS-ETOP) at 80 mg/m^2^ and 100 mg/m^2^, respectively, were given for six, 21-day cycles.

#### Radical prostatectomy

Prostatectomy was performed at 6–12 weeks post-chemotherapy. Clinical and laboratory assessments and were obtained 8–12 weeks post-prostatectomy. The decision to undergo aRT to the prostate bed/pelvis was based on the extent of adverse pathologic features (extraprostatic disease, positive surgical margins, high T stage or lymph node involvement) and the post-operative PSA.

#### Radiation

At 12–16 weeks following prostatectomy, aRT to the prostate bed/pelvis was given, as elected following surgery. In the same time frame, or immediately adjacent to aRT, SBRT was used to treat metastatic sites located outside of the adjuvant radiation field.

### Monitoring safety and secondary outcomes

Beyond the 8 weeks post-radiation visit, patients were followed and PSA levels were measured every 3 months. Testosterone levels were measured post-chemotherapy, post-radiation, and then every 3 months after completion of ADT. Imaging was performed at baseline and as clinically indicated.

Response to therapy was defined as PSA < 0.1 ng/mL post-radiation. If PSA was detectable, it was monitored monthly to confirm recurrence.

Adverse events (AEs) were graded using the National Cancer Institute Common Terminology Criteria for AEs (CTCAE) version 4.0. Toxicities recorded were ADT associated side effects, abiraterone-specific toxicities (liver dysfunction, hypertension, headaches, etc.), chemotherapy-associated neutropenia and NF (worst grade experienced), neuropathy and renal insufficiency, and radiation-associated toxicities. Post-surgical complications were graded using the Clavien-Dindo classification [20].

### Outcomes and data analysis

Patients were observed from initiation of chemotherapy until treatment failure (defined as detectable PSA at the completion of consolidative therapy) or the data closure date (3/5/2020).

Tracked outcomes included follow-up time (defined as the time from start of chemotherapy until the data closure date), 1-, 2- and 3-year undetectable PSA, defined as a PSA < 0.1 ng/mL at 1-, 2-, and 3-years from the start of ADT; normalization of testosterone (defined as ≥ 200 ng/dL after completion of ADT); biochemical recurrence (BCR) (the date of 2nd recorded PSA of 0.2 or greater, following nadir < 0.1); time to androgen independence (defined as failure of androgen deprivation therapy), and overall survival defined as the time from the start of ADT until death. As an observational report, all results are descriptive; continuous variables are expressed as medians with interquartile ranges (IQRs). Kaplan–Meier estimates were used for event-time distributions. All statistical analyses were done with STATA version 15.1 [21].

## Results

### Demographics

Twelve patients with newly diagnosed, untreated OMPCa initiated treatment between 10/17/2014 and 1/9/2017 (Table 1). Median age was 55.5 years (IQR 52.1–63.9), median ECOG was 0 (100%), 92% (11/12) were white, and median PSA was 14.7 ng/mL (IQR 6.5–33.7). Prostate biopsy pathology revealed adenocarcinoma in 92% (11/12) and adenocarcinoma mixed with SCC in 8% (1/12). Gleason score was 8–9 in 75% (9/12) of patients. 4/12 (33%) patients had a metastatic lymph node that was biopsy proven prostatic adenocarcinoma.

**Table 1:** Baseline demographics

Variable	No
Age, years	
Median (IQR)	55.5 (52.1–63.9)
Race	
White	11
Asian	1
Baseline PSA ng/mL	
Median (IQR)	14.7 (6.5–33.7)
Pathology	
Adenocarcinoma	11
Adenocarcinoma with small cell	1
Gleason	
6	1
7	2
8	4
9	5
Staging imaging	
^99m^Tc- bone scan	12
Positive	6
CT scan abdomen + pelvis	12
Bone	4
Lymph node	6
Bone and lymph node	1
Prostate only	1
PSMA ^18^F-DCFPyL PET/CT	7
Bone	3
Lymph node	1
Bone and lymph node	2
Prostate only	1
Extent of disease	
T1c	3
T2	5
T3b	4
N1 (pelvic LN)	4
N0	8
M0	1
M1a (non-regional LN)	3
M1b	8

*PSA* prostate -specific antigen, *PSMA *^*18*^*F-DCFPyL PET/CT* prostate specific membrane antigen targeted ^18^F-DCFPyL PET/CT scan, *LN* lymph node

### Staging

**Fig. 1:**
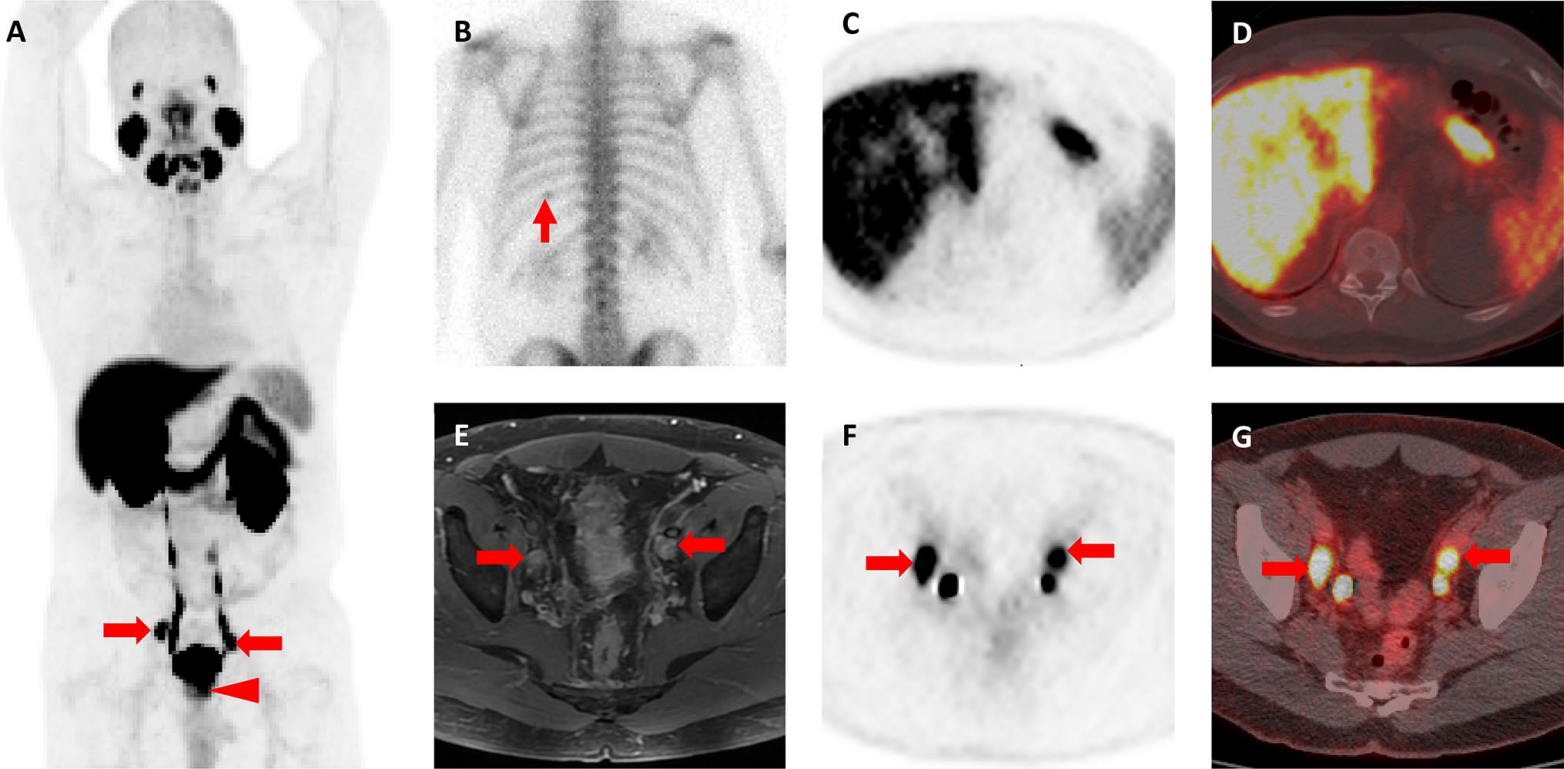
**a** Whole-body maximum intensity projection ^18^F-DCFPyL PET image demonstrates intense uptake in the patient’s primary tumor (red arrowhead) and bilateral pelvic lymph nodes (red arrows). No abnormal uptake is appreciated in the chest. **b** Posterior planar ^99m^Tc-methylene diphosphonate bone scan image shows abnormal uptake in the posterior left tenth rib (thin red arrow). This was considered suspicious in light of the known high-risk diagnosis. **c** Axial ^18^F-DCFPyL PET and **d**^18^F-DCFPyL PET/CT images show no evidence of abnormal uptake in the posterior left tenth rib. **e** Axial T1-weighted, post-contrast MRI of the pelvis demonstrates bilateral enlarged and enhancing pelvic lymph nodes (red arrows). **f** Axial ^18^F-DCFPyL PET and **g**^18^F-DCFPyL PET/CT show intense uptake in the bilateral pelvic lymph nodes (red arrows). The additional foci of uptake in (**f**) and (**g**) represent excreted radioactive urine in the distal ureters

100% (12/12) of patients had a ^99m^Tc-bone scan and a CT scan of the abdomen/pelvis. Most patients had either non-regional nodal (M1a) (25%, 3/12) or osseous (M1b) (67%, 8/12) metastases. The ^18^F-DCFPyL scan was intermittently available through several imaging protocols [22–24] and obtained when possible to complement conventional imaging (58%, 7/12) (Table 2). As compared to findings on conventional imaging, the ^18^F-DCFPyL scan modified patient staging: upstaged to OM (*n* = 1), down-staged to OM (*n* = 1) (Fig. 1), and identified (*n* = 5) and excluded (*n* = 4) metastatic sites.

**Table 2:** Staging for oligometastasis

Patient	Staging imaging						Radiation therapy		PSMA effect on treatment		Outcomes (through 3/5/20)		
	Conventional		PSMA PET										
	OM lesions		OM lesions						Metastatic sites				
	**#**	Location	#	Location	Staged into OM category?	Altered treatment?	aRT? SBRT?	# Mets treated	Added	Excluded	2-year PSA	3-year PSA	BCR
1	2 Bone + 1 LN	T-9, acetabulum + ext iliac LN	−	−	−	−	(+) SBRT	3	−	−	0	0	No
2	1 Bone	Sternum	3 bone	Sternum, T-9 vertebral body, right iliac bone		To include SBRT to 3 sites	(+) SBRT	3	y	n	0	0	No
3	1 Bone	Pubic ramus	2 bone	Pubic ramus-Right ilium) + left femur		To include SBRT to 2 sites	(+) SBRT	2	y	n	0	0	No
4	0	N/A	1 bone + 1 LN	1 Rib + 1 aortic bifurcation LN	Yes, upstaged	To include SBRT to 2 sites	(+) SBRT	2	y	n	0.3	N/A	Yes @ 25 mths
5	1 Bone	rib	−	−	−		(+) aRT; (+) SBRT	2*	−	−	0	0	No
6	2 Bone + ~ 5 LN	rib + scapula + pelvic sidewall, ext iliac and obturator LN’s (about 5)	1 bone + 3 LN	Ext and common iliac LN’s + scapula	Yes, downstaged	To include SBRT to fewer sites	(+) aRT; (+) SBRT	4	y	y	2.2	N/A	Yes @ 20 mths
7	2 Bone + 1 LN	9th rib, 11th rib, right pelvic sidewall LN**	0	Prostate only		To exclude SBRT to 2 bone lesions	(+) aRT	1	n	y		0	Yes @ 46 mths
8	2 Bone	10th rib + right iliac bone	2 bone	5th rib + iliac bone		To SBRT to 5th rib instead of 10th rib	( +) SBRT	2	y	y	0	0	No
9	4 LN	Para-aortic and internal iliac	−	−	−		( +) aRT; (+) SBRT	4	−	−	0	0.4	Yes @ 38 mths
10***	2 LN	Peri-prostatic + obturator	−	−	−		(+) aRT	2	−	−	0	0	No***
11	2 LN	Mesenteric + iliac bifurcation LN	1 LN	Iliac bifurcation LN		To exclude SBRT to mesenteric LN (was cold)	(+) SBRT	1	n	y	0	0.2	Yes @ 47 mths
12	4 LN	Retroperitoneal, iliac + pelvic sidewall	−	−	−		(+) aRT; (+) SBRT	4	−	−	0	0	No

*PSMA* prostate specific membrane antigen, *OM* oligometastases, *aRT* adjuvant radiation therapy, *SBRT* stereotactic body radiation therapy, *mets* metastases, *T-9* thoracic spine #9, *LN* lymph node

*Post-chemotherapy restaging imaging showed stable rib lesion and also 1 left iliac bone lesion; had SBRT to 2 bone lesions

**Lymph node was biopsy proven prostatic adenocarcinoma

***mixed adenocarcinoma and small cell carcinoma; followed with serial imaging (MRI abdomen/pelvis, CT chest, bone scans) with no evidence of recurrence

### Treatment details

#### Hormone therapy

**Fig. 2:**
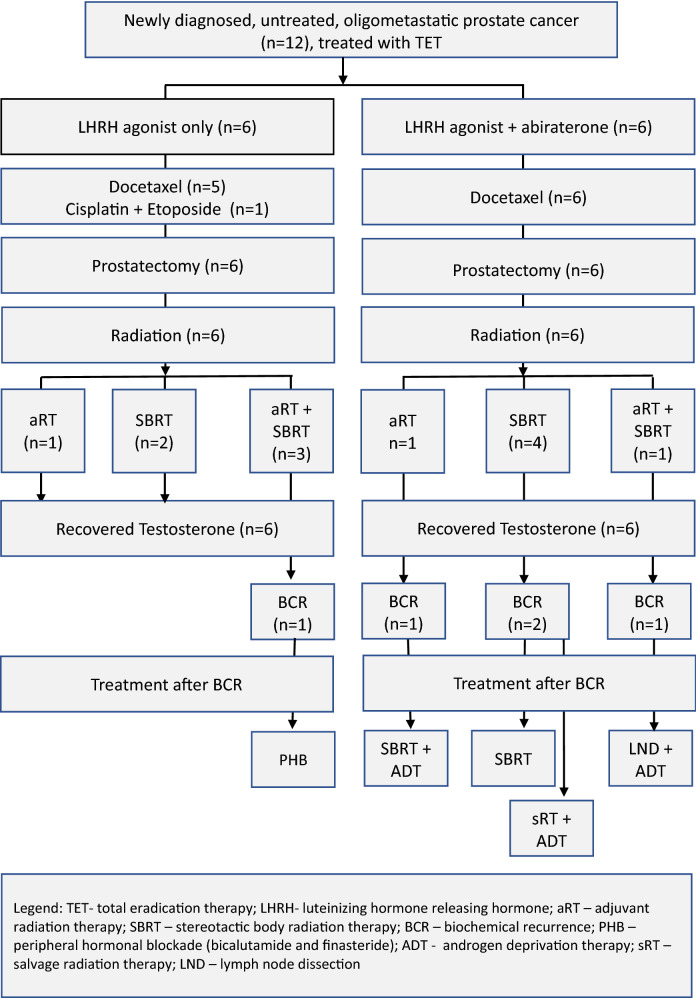
Flow chart of the prospective patient registry series of men with newly diagnosed, untreated oligometastatic prostate cancer, who underwent Total Eradication Therapy, with at least 2 years of follow-up

Treatment details are shown in Fig. 2. All patients were treated with an LHRH agonist, for either one year (83%, 10/12) or two years (17%, 2/12). Bicalutamide was given to 25% (3/12) of patients for a median duration of 4 weeks. Abiraterone was given concurrently with chemotherapy in 50% (6/12) of the patients for a median 2.8 months (IQR 2.0–3.1). Among patients who started ADT prior to chemotherapy, the median lead time was 22 days (IQR 21–26).

#### Chemotherapy

Median time to start chemotherapy was 0 days (IQR 0–21.5) after initiating ADT. Neoadjuvant docetaxel was given to 11 patients, total median dose 190 mg/m^2^, in 4 cycles, over a median 9 weeks (IQR 9–10). The initial docetaxel dosing regimen was used in 45% (5/11) of patients, whereas the revised regimen was used in 55% (6/11). The dosing regimens differed in total docetaxel given (median, 240 mg/m^2^ versus 180 mg/m^2^) within a similar time period (4 cycles within 9.5 versus 9 weeks). Neoadjuvant CIS-ETOP, total dose 480/600 mg/m^2^, was given in 6 cycles over 15 weeks to the patient with mixed adenocarcinoma and SCC. Median post-chemotherapy PSA was 0.2 (IQR 0–0.45). Four patients achieved an undetectable PSA prior to surgery.

#### Radical prostatectomy

All patients underwent a radical prostatectomy, a median 2.3 months (IQR 1.7–2.7) after the final chemotherapy dose. Pathologic findings revealed residual disease in all patients with a histologically apparent treatment effect in 67% (8/12), including significant treatment effects (33%, 4/12), partial treatment effects 17%, 2/12), and hormonal therapy effects (17%, 2/12); T2, T3a, and T3b disease was observed in 33% (4/12), 25% (3/12), and 42% (5/12), respectively, N1 disease was present in 67% (8/12), and positive surgical margins in 33% (4/12). Post-operative PSA was undetectable in 83% (10/12), and was not assessed in 17% (2/12) patients.

#### Radiation

Following prostatectomy, all patients underwent radiation therapy: adjuvant radiation to the prostate bed/pelvis only (2/12, 17%), adjuvant radiation to the prostate bed/pelvis and SBRT to metastatic sites outside of the adjuvant radiation field (4/12, 33%), and SBRT only (6/12, 50%). Adjuvant radiation to the prostate bed and pelvis was given at a median interval of 3.7 months (IQR 3.2–5.0) following prostatectomy. Post-radiation PSA was undetectable in all patients.

### Safety outcomes

#### Chemotherapy

Chemotherapy-related NF, grades 3/4, occurred in 18% (2/11) of patients; both were treated on the initial docetaxel dosing regimen. Neutropenia, grade 3, occurred in 45% (5/11) of patients: 3/6 treated in the initial dosing regimen (including the 2 patients with NF) and 2/5 treated in the revised dosing regimen. Chemotherapy-related renal insufficiency and peripheral neuropathy (both grade 1), occurred in the patient treated with CIS-ETOP.

#### Hormonal therapy

LHRH therapy-related grade 2 vasomotor symptoms, requiring medical therapy, occurred in 17% (2/12) of patients. Of the 6 patients given abiraterone, related toxicities occurred in 3/6, and included elevated alanine aminotransferase (grade 3), cough (grade 1) and fatigue (grade 3). In all cases, the toxicity improved or resolved with dose reduction or discontinuation of abiraterone.

#### Radical prostatectomy

Post-prostatectomy urethral strictures (grade IIIa, required dilation) occurred in 17% (2/12) of patients (ongoing, *n* = 1 and resolved, *n* = 1). No other complications were reported.

#### Radiation

Radiation-related toxicities during treatment occurred in 3/6 patients who underwent adjuvant radiation to the prostate bed/pelvis: grades 1–2 hemorrhoids (*n* = 3), grade 1 constipation (*n* = 2), nausea, vomiting, proctitis, urinary frequency/urgency (all grade 1, all *n* = 1); no long-term toxicities were reported.

Of the 6 patients who underwent SBRT only, 2/6 reported toxicities during treatment: grade 2 dysuria, diarrhea, hemorrhoids (all *n* = 1) and grade 1 diarrhea (*n* = 1); no long-term toxicities were reported.

### Treatment outcomes

Median follow-up was 48.8 months (IQR 43.6–60.6) (Table 3). There were no treatment failures. Overall survival was 100%. 1-, 2-, and 3YR undetectable PSA was 100% (12/12), 83% (10/12), and 67% (8/12), respectively. The median time to BCR was not reached. 5/12 (42%) patients had a BCR. Return of normal testosterone levels was achieved in all patients, at a median 18.9 months (IQR 15.7–22.2) when duration of hormone therapy was 1 year, and at 26.5 and 34 months when duration of therapy was 2 years. No patients progressed to androgen independence.

**Table 3:** Treatment outcomes

Time of follow-up, months	
median (IQR), *n* = 12	48.8 (43.6–60.6)
Return to non-castrate testosterone*, months	
ADT given for 1 year	
Median (IQR), *n* = 10	18.9 (15.7–22.2)
ADT given for 2 years	
* n* = 2	26.5 and 34
1 year undetectable PSA	12/12 (100%)
2 year undetectable PSA	10/12 (83%)
3 year undetectable PSA	8/12 (67%)
Time to biochemical recurrence, median	Not reached
Biochemical recurrence	5/12 (42%)

*ADT* androgen deprivation therapy, *PSA* prostate-specific antigen

*Non-castrate testosterone defined as ≥ 200 mg/dL serum testosterone level

Treatments administered following BCR included peripheral hormonal blockade (bicalutamide and finasteride) (*n* = 1), SBRT and ADT (*n* = 1), SBRT (*n* = 1), salvage RT and ADT (*n* = 1), and lymph node dissection and ADT (*n* = 1) (Fig. 2).

## Discussion

Men newly diagnosed with PCa and distant metastases have an overall 5 year survival rate of 31% [2]. There is an increasing appreciation that men with metastatic disease should be further classified by disease burden—either oligometastatic or polymetastatic—and that different treatment strategies should be developed for these clinical states. To treat men with lower metastatic disease burdens, we designed total eradication therapy (TET) to target all clinically evident and microscopic disease in men with newly diagnosed, untreated OMPCa. While our long-term objective is to determine if TET can improve survival in men with newly diagnosed OMPCa, in this study, our immediate objective was to determine if TET could be done safely. Our study population included 11/12 (92%) men with distant metastatic PCa, and 1/12 (8%) with surgical pathology significant for adenocarcinoma mixed with SCC. The median age of our population was 55 years: several studies have shown that men ≤ 55 years tend to have more aggressive disease than other age groups, outside of the ≥ 75–80 years age range [25, 26]. Here, we have demonstrated that once the docetaxel dosing was adjusted for a non-castrate population, TET was well tolerated and did not result in additive toxicities, in men with high grade, aggressive, OMPCa.

TET is an intensive treatment protocol given over 1 year, after which therapy is complete and testosterone is expected to recover. 67% (8/12) of the men treated with TET had a 3YR undetectable PSA, along with a recovered testosterone and freedom of ADT side effects. While patients treated with hormone therapy alone may experience an undetectable PSA at 3YR, it is expected that at a mean of 2–3 years on hormone therapy, androgen independence will occur [27], leaving the patient with a more aggressive cancer along with the cumulative side effects of hormone therapy. In contrast, despite 5/12 (42%) of the TET patients having had a BCR at 48 months median follow-up, 0/12 (0%) have transitioned to androgen independence.

OM remains a diagnosis of imaging, by virtue of the lone biomarker—the number of lesions – which traditionally has been assessed by bone scan and CT. The ^18^F-DCFPyL PET scan was recently shown to identify putative sites of disease in a majority of men in a prospective study of men with BCR. In that study, 68% (21/31) had at least one site of PSMA uptake consistent with a site of PCa, despite having negative conventional imaging [17]. In our population, the ^18^F-DCFPyL scan changed management by altering the eligibility for the diagnosis of OM, as well as by altering the number or location of metastases treated with SBRT.

Large, controlled trials [9, 28, 29], are conflicting on whether chemotherapy is indicated in patients with ‘low volume’ metastatic PCa. In these studies, the patients with ‘low volume’ disease had more advanced disease than our oligometastatic population, suggesting that adding in chemotherapy, when beyond curative intent, may not necessarily increase overall survival.

Although multiple studies are underway to optimize the treatment of men with newly diagnosed metastatic and OMPCa, they do not typically attempt to definitively treat all sites of clinically evident disease, as well as micrometastases. The addition of radiation to the primary tumor to ADT has been shown to increase survival [3]. SWOG 1802 [30] is recruiting men with newly diagnosed metastatic PCa to take part in a study of standard systemic therapy with or without definitive primary treatment. O’Shaughnessy and colleagues published a pilot study of multimodality treatment to definitively treat known sites of disease in early metastatic prostate cancer [14]. Twenty men with OM M1a (extrapelvic nodal disease) or M1b (bone disease) at diagnosis were treated with ADT, radical prostatectomy plus pelvic lymphadenectomy, and SBRT to osseous disease or the primary site. 20% of the patients had an undetectable PSA and non-castrate levels of testosterone at 20 months, suggesting that a multidisciplinary approach to treat all sites of clinically evident disease is worth exploring in men with OM disease at diagnosis. In our group of patients, more intense neoadjuvant therapy was given in the form of docetaxel and supracastration therapy with abiraterone. In addition, half of the men received aRT for residual disease post prostatectomy. Our rationale was that TET, if it could be given safely, and given at the earliest stage of metastatic disease, would result in better outcomes, i.e. as close as is possible to total eradication of disease.

Our registry study of patients with newly diagnosed, untreated OMPCa, treated with TET, served as a model to inform the optimal study design for prospective TET trials. We then initiated two prospective phase II clinical trials (protocols available in eSupplements) to assess the efficacy of TET in men with newly diagnosed OMPCa (< 5 sites of metastases). The first trial, TED-1 (total eradication of disease), is for treatment naïve men with OMPCa. The second trial, TED-2, is for men diagnosed with OMPCa within 6 months of prostatectomy. The TED-1 study design includes neoadjuvant treatment encompassing up to 6 months of androgen deprivation and up to 6 cycles of docetaxel with concurrent abiraterone and prednisone. Following docetaxel therapy, patients with a PSA response of at least a 50% decrease from baseline, proceed to maximum consolidative therapy. Consolidative therapy includes definitive local therapy with radical prostatectomy (RP), ± adjuvant radiation therapy (RT) (given in the setting of adverse pathologic features), ± consolidative stereotactic body radiation therapy (SBRT) to the oligometastatic sites. Androgen deprivation is continued through consolidative therapy; it is given for 1 year in total. The TED-2 study design, for men post-prostatectomy (< 6 months), includes adjuvant treatment encompassing up to 6 months of androgen deprivation and up to 6 cycles of docetaxel with concurrent abiraterone and prednisone. Following docetaxel therapy, patients proceed to maximum consolidative therapy. Consolidative therapy includes ± adjuvant radiation therapy (RT) (given in the setting of adverse pathologic features) ± consolidative stereotactic body radiation therapy (SBRT) to the oligometastatic sites. Androgen deprivation therapy is continued through consolidative therapy; it is given for two years in total. Follow-up for TED-1 and TED-2 is for 2 years after completion of ADT treatment and includes serial labwork and imaging. The primary endpoint is 2- and 3-year progression-free PSA (< 0.2 ng/mL), respectively.

Potential toxicities associated with the individual modalities of TET include post-chemotherapy neutropenia and febrile neutropenia, and post-prostatectomy or radiotherapy non-neutropenic toxicities including urinary incontinence, urinary complications requiring instrumentation, rectal or anal complications requiring instrumentation, and secondary malignancy. To mitigate potential additive toxicity from TET multimodal therapies, the TED 1 and 2 protocols include early stopping boundaries. Based on prior probabilities of the average risks of toxicities, the early stopping boundaries incorporate safety monitoring after each patient, and outline when to stop the studies should the threshold of acceptable toxicity be crossed.

## Conclusions

**Fig. 3:**
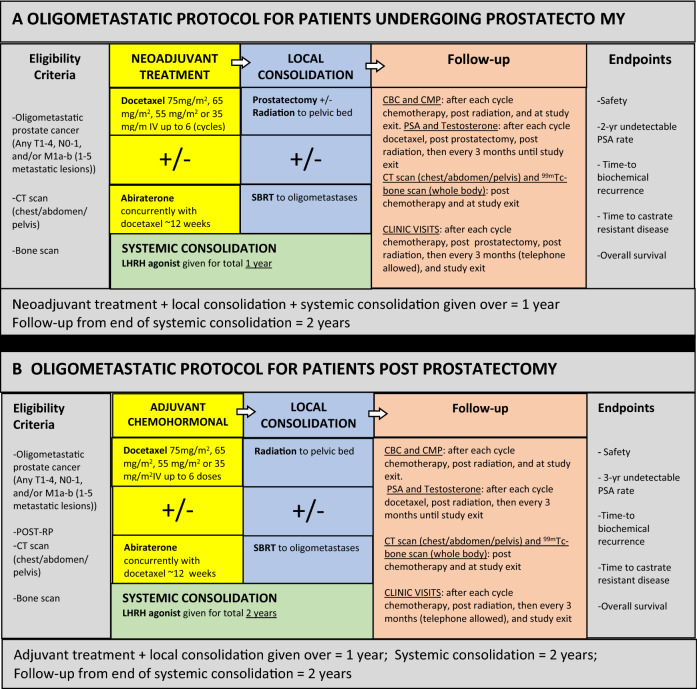
Following the prospective patient registry series of men with newly diagnosed, untreated oligometastatic prostate cancer (OMPCa), who underwent Total Eradication Therapy (TET), two clinical trials were initiated utilizing TET in men with newly diagnosed OMPCa, both prior to prostatectomy (NCT02716974) and post-prostatectomy (NCT03043807). **a** Study schema-A phase II study of definitive therapy for newly diagnosed men with oligometastatic prostate cancer, NCT02716974. **b** Study schema-A phase II study of definitive therapy for newly diagnosed men with oligometastatic prostate cancer after prostatectomy, NCT03043807

Tracking the treatment and outcomes of a registry study of patients with newly diagnosed, untreated OMPCa allowed us to assess the impact of TET. Our outcomes suggest that multi-modality therapies can be combined safely resulting in durable responses. Therefore, we have since initiated clinical trials utilizing TET in men with newly diagnosed OM disease, both prior to prostatectomy (NCT02716974 [31]) and post-prostatectomy (NCT03043807 [32]) (Fig. 3).
